# The nutritional landscape of host plants for a specialist insect herbivore

**DOI:** 10.1002/ece3.5730

**Published:** 2019-11-08

**Authors:** Jerome Keaton Wilson, Laura Ruiz, Jesse Duarte, Goggy Davidowitz

**Affiliations:** ^1^ School of Natural Resources and the Environment University of Arizona Tucson AZ USA; ^2^ Neuroscience and Cognitive Science University of Arizona Tucson AZ USA; ^3^ Ecology and Evolutionary Biology University of Arizona Tucson AZ USA; ^4^ Department of Entomology University of Arizona Tucson AZ USA

**Keywords:** geometric framework, *Manduca sexta*, nutrient space, plant nutrition, plant‐insect interactions

## Abstract

1. Nutrition has far‐reaching effects on both the ecology and evolution of species. A substantial body of work has examined the role of host plant quality on insect herbivores, with a particular focus on specialist–generalist dynamics, the interaction of growth and other physiological attributes on fitness and tritrophic effects. Measures of plant quality usually involve one or two axes of nutritional space: typically secondary metabolites or elemental proxies (N and C) of protein and carbohydrates, respectively.
2. Here, we describe the nutrient space of seven host plants of the specialist insect herbivore, *Manduca sexta*, using an approach that measures physiologically relevant sources of nutrition, soluble protein and digestible carbohydrates. We show that plant species differ markedly in their nutrient content, offering developing insect herbivores a range of available nutrient spaces that also depend on the age of the leaves being consumed.
3. The majority of host‐plant species produce diets that are suboptimal to the herbivore, likely resulting in varying levels of compensatory feeding for *M. sexta* to reach target levels of protein to ensure successful growth and development. Low‐quality diets can also impact immune function leading to complex patterns of optimization of plant resources that maximizes both growth and the ability to defend from parasitoids and pathogens. This study is the first to quantify the nutrient space of a suite of host plants used by an insect herbivore using physiologically relevant measures of nutrition.

## Graphical abstract

The nutritional landscape of host plants available to a specialist insect herbivore is varying, with particular emphasis on physiologically relevant measures of nutrition.

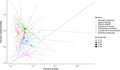

## 1 INTRODUCTION

Nutrition shapes broad patterns in ecology and evolution and is particularly important in impacting the interactions between plants and insect herbivores. Inter‐ and intraspecific variation in nutrition can impact herbivore fecundity (Awmack & Leather, 2002), shape host plant and generalist–specialist dynamics (Behmer & Joern, 2008; Coley, Bateman, & Kursar, 2006; Sznajder & Harvey, 2003), and can affect population and community dynamics (Denno et al., 2000; Joern, Provin, & Behmer, 2012). Though we are beginning to understand that nutritive components of plants can interact with each other and with secondary metabolites to affect insect performance and fitness (Chen, 2008; Cotter, Simpson, Raubenheimer, & Wilson, 2010; Simpson & Raubenheimer, 2001; Wilson, Ruiz, & Davidowitz, 2019) and that these effects can cascade upward to higher trophic levels (Sznajder & Harvey, 2003), in many systems we still lack a basic understanding of the nutritional landscape offered to insect herbivores over different spatial and temporal scales.

Quantifying nutritional quality of different diet sources can be challenging, as diets are multifaceted. Organisms must balance main nutritive components that are required to generate energy (carbohydrates, protein, and lipids) with each other, but also with micronutrients and the presence of secondary metabolites that may be detrimental to performance. The Geometric Framework (GF) is a conceptual framework that defines a given diet in multidimensional space, where each axis represents the level of an individual component, which can include macro or micronutrients, and even secondary metabolites (Simpson & Raubenheimer, 2001). This framework has been widely used to examine a variety of different topics in nutritional ecology, from target intake rates (Raubenheimer, Simpson, & Mayntz, 2009), behavior (Raubenheimer & Simpson, 1993), and optimal foraging theory (Simpson, Sibly, Lee, Behmer, & Raubenheimer, 2004). Here, we use the GF to explore nutrient space across host‐plant species and plant tissues on two important axes (Behmer, 2009; Deans, Behmer, Fiene, & Sword, 2016): plant soluble protein and digestible carbohydrates.

Elemental analysis of plants has long been the standard for quantifying protein and carbohydrate components with nitrogen acting as a proxy for protein and carbon acting as a proxy for carbohydrates (Berner, Blanckenhorn, & Körner, 2005; Deans et al., 2016; Telang, Booton, Chapman, & Wheeler, 2001). Though this approach has generated useful research outcomes (Joern et al., 2012), researchers have recently made the case for using more direct measurements of soluble protein and digestible carbohydrates (Deans et al., 2016). This argument stems from the fact that some percentage of carbon and nitrogen atoms exist in non‐nutritive compounds in plant tissue (i.e., cellulose, lignin, and allelochemicals), making linkages from elements to nutritive biochemicals difficult. Though current methods (Deans et al., 2018) do not account for species‐specific differences in gut biochemistry, they likely make better proxies for nutrient content than traditional elemental analysis (Deans et al., 2018). Here, we take an insect‐centric approach and use methods outlined by recent work (Deans et al., 2016) to examine soluble protein and digestible carbohydrates in a suite of host plants used by a specialist insect herbivore: *Manduca sexta* (Sphingidae).

*Manduca sexta* has a wide distribution across tropical and temperate regions of the Nearctic and is classified as a specialist herbivore—feeding on a variety of host plants within Solanaceae (Yamamoto & Fraenkel, 1960), but has also adopted a nonsolanaceous host (*Proboscidea* spp., Martyniaceae) (Mechaber & Hildebrand, 2000) (Table 1). Female *M. sexta* moths deposit eggs on host plants during the monsoon season in southeastern Arizona. Larvae develop through five instars and eventually descend to the ground where they bury themselves, construct a pupal chamber, and either emerge as adults in approximately 3 weeks or enter diapause and emerge the following season. The costs and benefits of the use of different host plants have been examined in terms of performance (Diamond & Kingsolver, 2011), fitness (Diamond & Kingsolver, 2010), and predator avoidance (Mira & Bernays, 2002), but to our knowledge, the nutritional landscape of the range of host plants available has yet to be explored, despite strong evidence for interactions between nutritional components and performance, fecundity, and immune function (Diamond & Kingsolver, 2010, 2011; Wilson et al., 2019).

**Table 1:** Common host plants of *Manduca sexta* across its range

Plant species	Common name	Famliy	Geographic region	Citation	Used in this study
*Capsicum annuum*	Pepper	Solanaceae	Widespread across US	Madden and Chamberlin, (1945)	Yes
*Datura discolor*	Jimsonweed	Solanaceae	Southwestern US	Reisenman et al., (2013)	Yes
*Datura wrightii*	Jimsonweed	Solanaceae	Southwestern US	Mira and Bernays, (2002)	Yes
*Lycopersicon esculentum*	Tomato	Solanaceae	Widespread	Ashmead, (1887), Yamamoto and Fraenkel (1960)	Yes
*Nicotiana attenuata*	Cultivated tobacco	Solanaceae	Southwestern US	van Dam, Hadwich, and Baldwin (2000)	Yes
*Nicotiana tabacum*	Coyote Tobacco	Solanaceae	Widespread across US	Madden and Chamberlin, (1945)	Yes
*Physalis angulata*	Groundcherry	Solanaceae	Southeastern US	Madden and Chamberlin, (1945)	No
*Probiscidea louisianica*	Devil's Claw	Martyniaceae	Southwestern US	Mechaber and Hildebrand, (2000)	No
*Probiscidea parviflora*	Devil's Claw	Martyniaceae	Southwestern US	Mechaber and Hildebrand, (2000)	Yes
*Solanum carolinense*	Horsenettle	Solanaceae	Southeastern US	Madden and Chamberlin, (1945)	No
*Solanum nigrum*	Black nightshade	Solanaceae	Southeastern US	Madden and Chamberlin, (1945)	No
*Solanum tuberosum*	Potato	Solanaceae	Southeastern US	Madden and Chamberlin, (1945)	No

Here, we use seven host plants that are commonly used by *M. sexta* across its range that span cultivated and wild species and the two main families (Solanacae and Martyniaceae) of plants fed upon by *M. sexta*. Plants were grown under common‐garden conditions in an experimental greenhouse to address three main questions: (a) how much intra‐ and interspecies variation is there in the nutritional space of different host plants used by *M. sexta*, (b) how much variation is there in tissue quality within a given plant (i.e., are there differences between old and young leaves), and (c) how do the nutritional landscapes of different host plants relate to previous work examining growth and immune function in modified nutritional landscapes of artificial diets. To our knowledge, this work is the first to examine ecologically relevant (soluble protein and digestible carbohydrate) nutrient space across a suite of host plants for a given species, and only one of a few to use these nutrient assays to show the insect‐centric nutrient space using the geometric framework (Deans et al., 2016; Li, Volenec, Joern, & Cunningham, 1996; Ojeda‐Avila, Woods, & Raguso, 2003; Sánchez, Rivero, Ruiz, & Romero, 2004; Stieger & Feller, 1994). By examining patterns of variation in nutrition across a wide range of host plants, we gain deeper insight into the ecological and evolutionary dynamics that shape fundamental relationships between plants, herbivorous insects, and third trophic levels.

## 2 METHODS

### 2.1 Plant cultivation and material collection

Plants were germinated from seed in late May of 2018. Seed of *Datura wrightii*,* Datura discolor*, and *Proboscidea parviflora* were wild collected from populations in southern Arizona. Seed for *Nicotiana attenuata*,* Nicotiana tabacum* (Brightleaf; New Hope Seed Company), *Capsicum annuum*, and *Solanum lycopersicum* (Atlee Burpee and Company) was obtained from commercial sources. Seedlings were transplanted in two batches between late May and early June of 2018 into cylindrical 1‐gallon (~3.78 L) or 2‐gallon pots (~7.57 L) (previous unpublished work has shown that *D. wrightii* grows better in larger pots) using a soil mixture of Sunshine Propagation Mix #3, 20‐grit sand, and Vermiculite (Therm‐O‐Rock) in a 3:1:2 ratio. Plants were kept in a temperature‐controlled greenhouse at the University of Arizona, Tucson, Arizona (between 21 and 35°C temperature on a natural light cycle for this time of year). The plants were watered *ad libidum* no less than four times a week. At 33–42 days of growth, two leaf samples were harvested from plants—the oldest intact leaf from low on the stem and a younger, smaller leaf (or leaves, depending on the size—to provide enough leaf tissue for effective nutrient assays) growing at the top of the plant. Plant age was recorded as the difference in time between first transplant (from germination tray into individual containers) and when leaf samples were sampled. We recorded the height of each plant and whether or not the plant had flowers or fruit, to control for these factors in subsequent analyses. Samples were then placed in labeled, 50 ml, conical Eppendorf tubes and put into a transport container that was filled with ice. The containers were taken to a laboratory on the University of Arizona campus where the tubes were removed and placed into a −80°C freezer for storage. In total, we collected samples from 82 plants (between 9 and 14 individuals of each species with a sample from old and young leaf tissue for each plant) with 164 samples in total.

### 2.2 Lyophilization and material processing

Our protocol for sample preparation and analysis is adapted from work by Deans et al. (2018). After reaching −80°C, samples were taken out of the freezer and placed in two Eppendorf tube trays. The trays of samples were stacked into the main chamber of a lyophilizer (Virtis Freezemobile 6) where they were processed for a minimum of 48 hr, at a pressure between 100 and 150 Millitorr and a temperature of −40°C. After lyophilization, samples were stored at room temperature. The shelf‐stable samples were then preground using a ceramic mortar and pestle and then placed into a Mills grinder that was set to run on the highest level of grinding for 30 s.

### 2.3 Soluble protein and digestible carbohydrate assays

#### 2.3.1 Soluble protein

We measured 20 mg of lyophilized leaf tissue in duplicate into 1.5 ml Eppendorf tubes and added 500 μl of 0.1 M NaOH. We then sonicated tubes for 30 min (Branson 2510, Branson Ultrasonics) and placed tubes in a 90°C water bath (VWR 5L Avantor) for 15 min. Tubes were then centrifuged for 10 min at 13,000 rpm (7,558 *g*, VWR Galaxy 16D, Avantor), and the supernatant transferred to another 1.5 ml Eppendorf tube. We pipetted 300 µl of 0.1 M NaOH to the Eppendorf tube with the pellet and centrifuged for another 10 min at 13,000 rpm (7,558 g). Then, we transferred the supernatant to the other Eppendorf tube and added 11 µl of 5.8 M HCl. We vortexed tubes for a few seconds each, so HCl would distribute evenly throughout the sample and measured the pH of three randomly chosen samples (out of a batch of 4–8, depending on the run) to ensure that we had achieved a neutral pH. We pipetted 90 µl of 100% TCA solution into samples and placed them on ice for 30 min, to facilitate protein precipitation. We then centrifuged samples again (at 13,000 rpm for 10 min), removed the supernatant, and washed pellets with 100 µl of −20°C acetone. Acetone was removed and samples were allowed to air‐dry in a 15°C refrigerator for approximately 10–20 min. Samples were visually inspected to ensure that excess acetone had evaporated, after which protein pellets were resuspended with 1 ml of 0.1 M NaOH. This process was facilitated by vortexing, placing samples back in the 90°C hot water bath, and by adding 20–40 µl of one normal NaOH when needed. Samples were then diluted by mixing 50 µl of the resuspended solution to 950 µl of deionized water. We then added 60 µl of each new diluted solution to a 96‐well plate in triplicate (to generate technical replicates of each sample—these were subsequently averaged to generate the raw values for further analyses) and added 100 µl of deionized water to each sample wells. Between 4 and 8 individual leaf samples (in triplicate) were run per batch. We also added 160 µl of IgG standard solutions (0–12.16 µg of protein across six samples) in triplicate to the plate. A total of 40 µl of BioRad protein assay dye reagent was added to each well, and the entire plate was incubated at 23°C for 5 min. Absorbance was measured at 595 nm in a spectrophotometer (Thermoscientific Multiskan GO, Thermo Fisher Scientific), and we used 2nd‐order polynomial regressions for soluble protein standard curves because these models provided better fits than linear models.

#### 2.3.2 Digestible carbohydrates

We measured 20 mg of lyophilized leaf tissue and added them to glass vials with 1 ml of 0.1 M H_2_SO_4_ in duplicate. Vials were placed in a water bath at 100°C (a beaker with deionized water on a hot plate) for an hour. After samples cooled, we transferred them from the glass vials to 1.5 ml Eppendorf tubes and centrifuged them for 10 min at 13,000 rpm. We transferred 15 µl of supernatant from each sample to glass test tubes with 385 µl of deionized water each; we also prepared six glass test tubes containing 0–0.1875 µg/µl glucose (our standard curve). We added 400 µl of 5% phenol to test tubes and pipetted 2 ml of concentrated H_2_SO_4_ (95%–98%) onto the surface of the solution. Test tubes were allowed to sit for 10 min, after which we vortexed them on a low setting and allowed them to sit for another 30 min. Finally, 1 ml of each sample and standard was transferred to cuvettes and run in duplicate on the spectrophotometer at 490 nm. We used linear regression models for digestible carbohydrate standard curves.

### 2.4 Data analysis

All analyses were performed in R (http://www.r-project.org; 3.5.0 ‘Joy in Playing). Ten of 328 (analysis of carbohydrates or protein from 164 leaf samples) samples generated extreme outliers with values outside of the range of biologically realistic levels (>75% or <0% protein or carbohydrates by weight) were removed prior to analyses and were likely the result of errors in protein or carbohydrate analysis. Percentages of digestible carbohydrates and soluble protein were logit‐transformed before statistical analysis to conform to normality assumptions. We used a series of linear mixed effects models (*lme4* and in R; Bates, Sarkar, Bates, & Matrix, 2007) to determine the effects of species and leaf age (as fixed effects) on the soluble protein and carbohydrate contents of leaves while controlling for the fact that we sampled two leaves (young and old) on a single plant and controlling for the time interval between germination and leaf harvest (random effects). During model formulation, this modeling scheme generated some models with singular fit, so we used a simpler model (as per R package recommendations) by removing the time interval between germination and leaf harvest. We used simplified models when determining the relationship between species and age on protein:carbohydrate (p:c) ratio and for percentage carbohydrates. Model estimates were similar between singular fits and the simpler model we report. Overall effects of fixed variables were generated using a Type 2 analysis of variance (ANOVA) using Satterthwaite's degrees of freedom method (Kuznetsova, Brockhoff, & Christensen, 2017; Satterthwaite, 1946). We used a similar approach when determining what fixed effects had impacts on nutritional content and protein to carbohydrate ratios. To generate *F*‐statistics and *p*‐values from linear effects models in *lmer*, we used the *lmerTest* package (Kuznetsova et al., 2017) to generate Type 2 analysis of variance (ANOVA) with Satterthwaite's degrees of freedom method.

## 3 RESULTS

### 3.1 Soluble protein and digestible carbohydrate content

**Figure 1:**
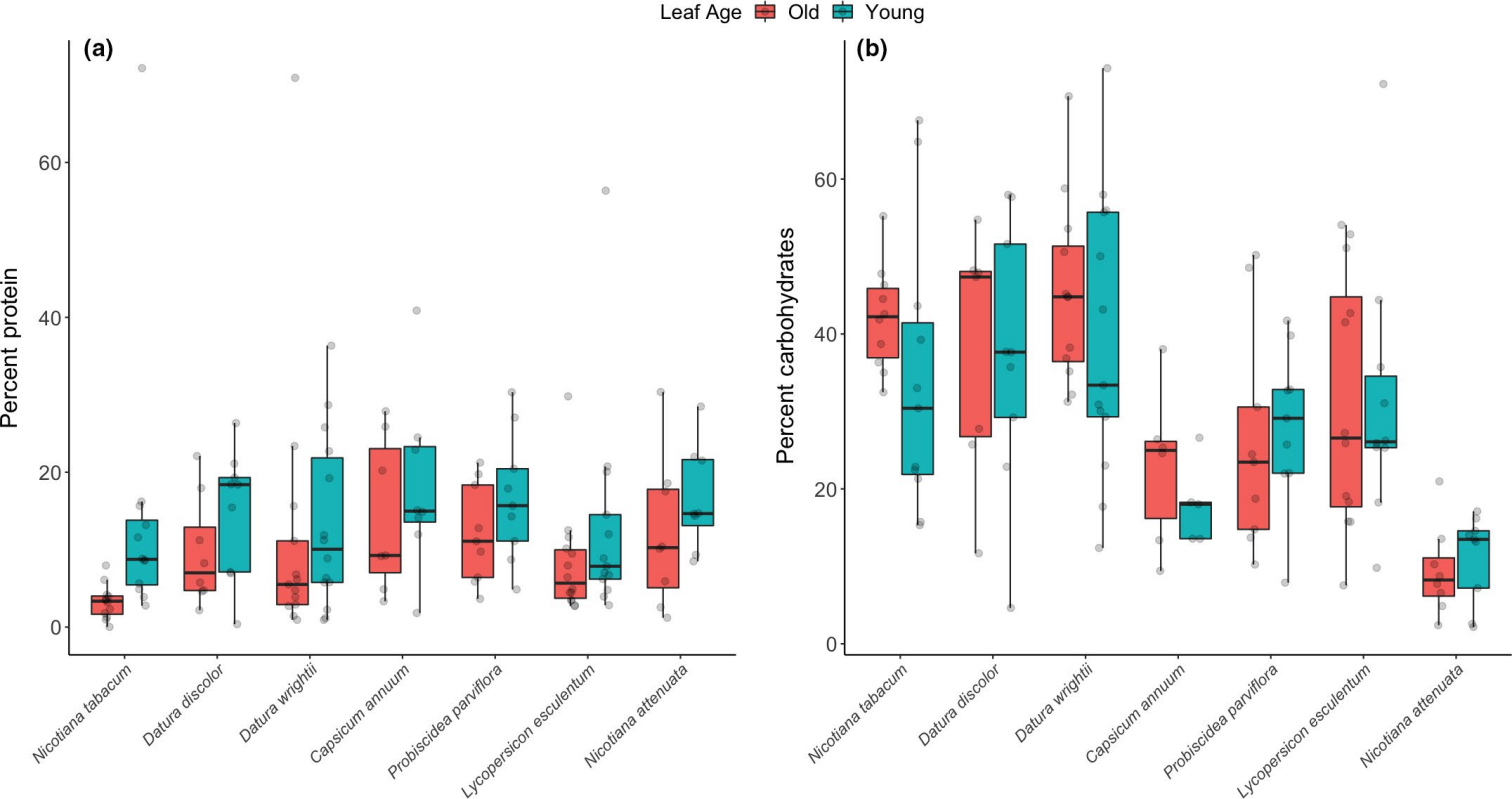
Percent soluble protein (a) and digestible carbohydrates (b) in old and young leaves across seven host plants of *Manduca sexta*. Different leaf ages are coded by colored box and whisker plots (red boxes are old leaves and blue boxes are young leaves). Each horizontal bar represents the median of each group, and solid boxes span the interquartile range and the whiskers extend to the lowest and highest values (excluding outliers). Raw values are represented by jittered transparent points

Host‐plant species differed significantly in both the percentage of soluble protein (*F* = 3.3943, NumDF = 6, DenDF = 41.666, *p* = .008; Figure 1a; Table 2) and digestible carbohydrate (*F* = 11.841, NumDF = 6, DenDF = 66.350, *p* < .0001; Table 3) after controlling for the time period between when a plant was first transplanted (for protein – we didn't control for this in carbohydrates because of issues with singular fits in model generation) and when we sampled leaves (Table 2). Plants species differed more in digestible carbohydrate content than in soluble protein content, and also had higher variance (Figure 1). *N. attenuata* had the lowest amount of soluble carbohydrates of all species with an average of 10.3 ± 6% while *D. wrightii* had the highest with an average of 43.5 ± 15% (Figure 1a). Additionally, *N. tabacum* had low levels of soluble protein with an average of 6.9 ± 5% while *C. annuum* had the highest at 15.6 ± 9.7%.

**Table 2:** Linear mixed‐effects model of logit‐transformed protein content across species and leaf age

Parameter	Estimate	*SE*	*df*	*t*‐value
Fixed effects				
Intercept	−3.1446	0.2767	31.4166	−11.365
*D. discolor*	0.5657	0.3273	50.5021	1.728
*D. wrightii*	0.1787	0.2991	45.7715	0.597
*C. annuum*	0.7599	0.3748	39.7665	2.027
*P. parviflora*	1.2276	0.3223	45.1209	3.808
*L. esculentum*	0.2264	0.3264	45.9059	0.693
*N. attenuata*	0.7797	0.4761	45.2963	1.638
Age	0.3849	0.1635	48.7966	2.354

Groups	Variance	*SD*
Random effects		
Individual plant	0.00453	0.0673
Plant age	0.0843	0.2904
Residual	0.63680	0.7980

Parameter	Sum square	NumDF	DenDF	*F*	*p*
ANOVA (Type II Satterthwaite)					
Species	12.969	6	41.666	3.3943	.0081
Age	9.4592	1	48.797	5.5434	.0226

**Table 3:** Linear mixed‐effects model of logit‐transformed digestible carbohydrate content across species and leaf age

Parameter	Estimate	*SE*	*df*	*t*‐value
Fixed effects				
Intercept	−0.4818	0.1909	73.5939	−2.524
*D. discolor*	−0.1424	0.2727	68.7730	−0.522
*D. wrightii*	0.2076	0.2466	64.2282	0.842
*C. annuum*	−0.8540	0.3105	64.0387	−2.750
*P. parviflora*	−0.5466	0.2705	60.8049	−2.020
*L. esculentum*	−0.4078	0.2511	68.4308	−1.624
*N. attenuata*	−1.7974	0.2731	62.6930	−6.581
Age	−0.1135	0.1027	60.5893	−1.105

Groups	Variance	*SD*
Random effects		
Individual plant	0.1947	0.4413
Residual	0.3255	0.5706

Parameter	Sum sq	NumDF	DenDF	*F*	*p*
ANOVA (type II Satterwhaite)					
Species	23.1294	6	66.350	11.841	<.0001
Age	0.3978	1	60.589	1.222	.2733

Leaf age had significant effects on soluble protein (*F* = 5.5434, NumDF = 1, DenDF = 48.797, *p* = .0226) but not digestible carbohydrate content (*F* = 1.222, NumDF = 1, DenDF = 60.589, *p* = .2733). Effects on protein content were consistent across plant species, with young leaves having higher levels of soluble protein compared with old leaves (Figure 1a).

**Figure 2:**
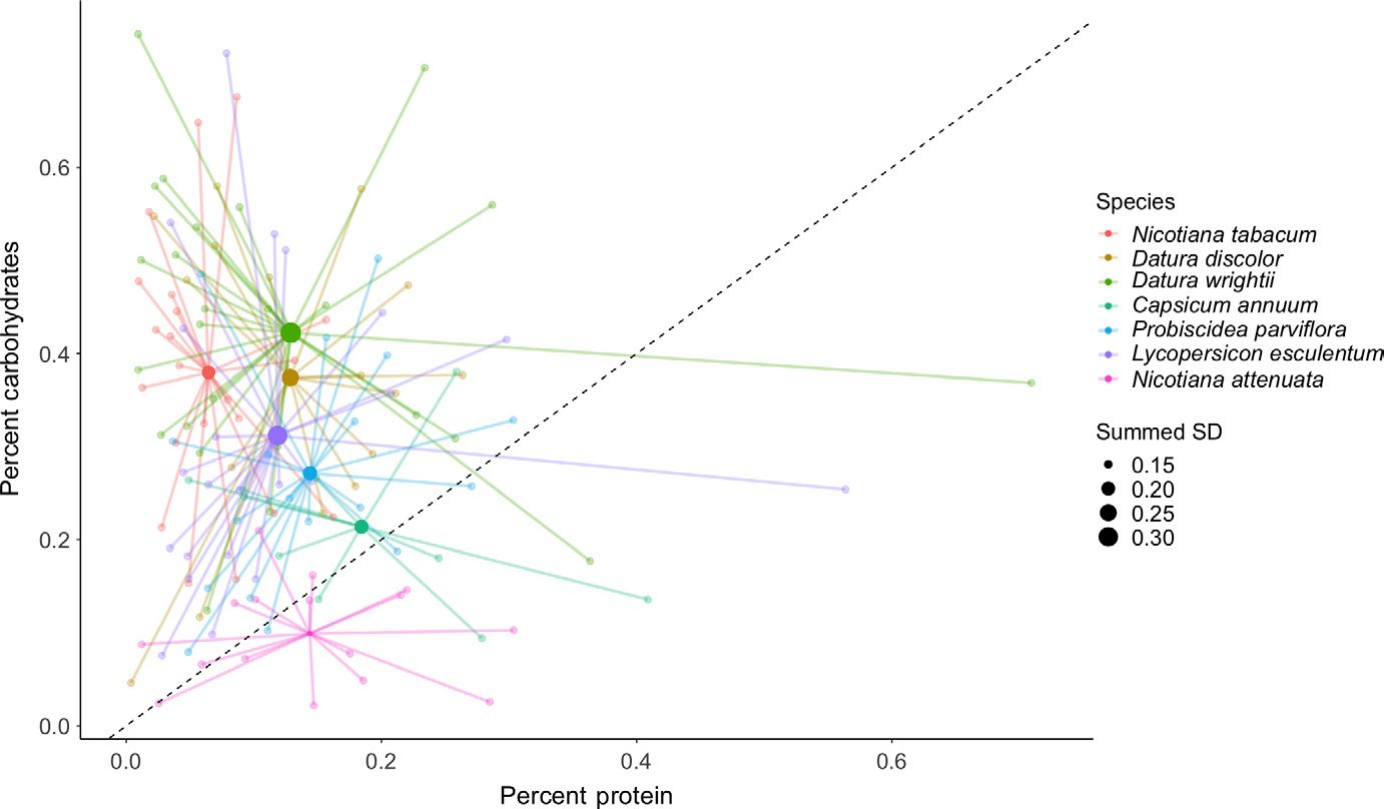
Host plant position in nutrient space. The raw values of soluble protein and digestible carbohydrate content (percent by weight) of each host plant species are represented by small points color coded by species ‐ young and old leaves are combined in this figure. Larger colored points represent the centroid for each group, with their size being scaled to the summed standard deviation for each axis (a larger centroid point indicates more variance in nutrient space for a given species). The diagonal dotted line represents a 1:1 ratio between carbohydrate and protein content

Though there was significant variation among individual plants in both carbohydrate and protein content, there were consistent differences among species in nutrient space. The biggest separation between species was in the percentage of carbohydrates, with *N. attenuata* showing the clearest distinction among other species in nutrient space (Figure 2), notably because of its decreased percentage of digestible carbohydrates.

**Figure 3:**
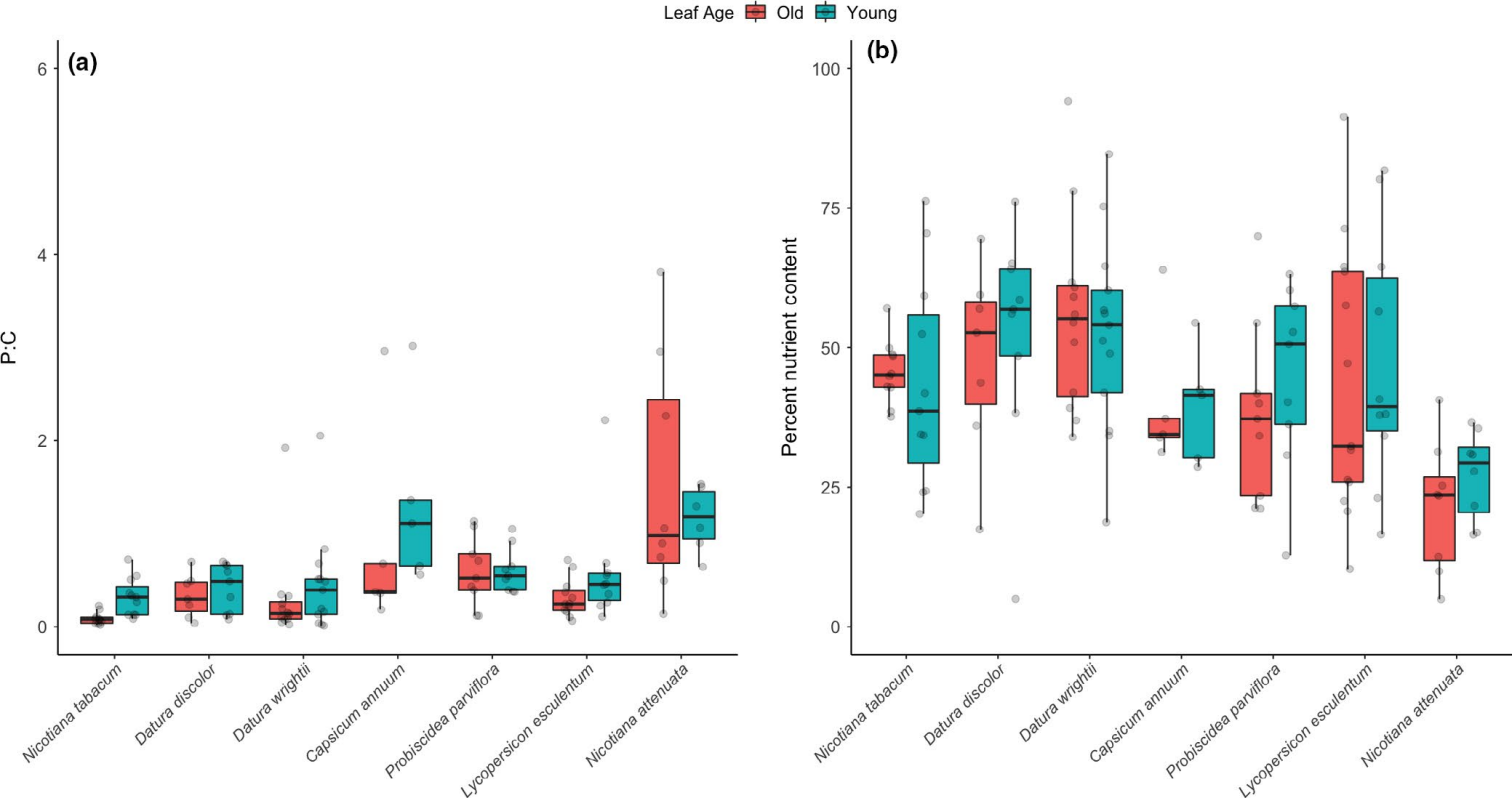
Soluble protein to digestible carbohydrate ratio (a) and percent nutritive content (by weight) (b) in old and young leaves across seven host plants of *Manduca sexta*. Different leaf ages are coded by colored box and whisker plots (red boxes are old leaves and blue boxes are young leaves). Each horizontal bar represents the median of each group, and solid boxes span the interquartile range and the whiskers extend to the lowest and highest values (excluding outliers). Raw values are represented by jittered transparent points

We also combined measurements of carbohydrates and protein to determine the protein:carbohydrate (p:c) ratio for each species across different leaf ages. Species had a significant effect on p:c (*F* = 3.0773, NumDF = 6, DenDF = 76.231, *p* = .009) but leaf age did not (*F* = 1.2090, NumDF = 1, DenDF = 73.752, *p* = .275). On average, young leaves had higher p:c ratios than old leaves (mostly due to higher protein levels; Figure 1a), and *N. attenuata* had the highest p:c ratios of any species in both old and young leaves, in spite of large amounts of variation among individuals (Figure 3a). Additionally, we also quantified the nutrient content of a given sample—the percentage by dry weight of a given leaf that is soluble protein and digestible carbohydrate (the remaining weight is non‐nutritive). Species differed significantly in nutritive content (*F* = 4.1724, NumDF = 6, DenDF = 33.655, *p* = .003; Figure 3b) with *N. attenuata* having the lowest average percentage (24.7 ± 9.7%) and *D. wrightii* having the highest (56.7 ± 19.1%). Leaf age had no effect on nutritive content (*F* = 0.0335, NumDF = 1, DenDF = 37.645, *p* = .856).

## 4 DISCUSSION

The importance of interacting nutritional components of diet has long been recognized as a fundamental factor that shapes the ecology and evolution of many species, including insect herbivores and their host plants. However, only a handful of papers have focused on measuring these values from an insect‐focused nutritional perspective (Deans et al., 2016, 2018; Li et al., 1996; Machado, Arce, Ferrieri, Baldwin, & Erb, 2015; Sánchez et al., 2004; Stieger & Feller, 1994)—soluble protein and digestible carbohydrates are important nutritional components because they are better representation than elemental measurements of the nutritional content available from a given diet and minimize the impact of carbohydrates or protein that are present in plant tissue but are non‐nutritive to an herbivore such as cellulose or lignin. Some of our results follow similar patterns to those demonstrated in other plant species: (a) Younger leaves typically have a higher protein:carbohydrate ratio (p:c) than older leaves (Deans et al., 2016; Li et al., 1996; Machado et al., 2015; Sánchez et al., 2004; Stieger & Feller, 1994); Figure 3), and (b) there can be significant variation among individuals within a species in both digestible carbohydrate and soluble protein (Deans et al., 2016; Machado et al., 2015). Both of these patterns have strong implications for insect herbivores including the evolution of behavioral strategies of female oviposition choice and larval feeding (potential selection for insects being able to predict leaf quality and tailor feeding behavior appropriately), and the importance of phenology and among‐ and within‐plant nutritional heterogeneity in structuring herbivore‐plant interactions (Bernays & Chapman, 1994). However, one of our main results is striking: None of the common host plants we measured provide p:c ratios for *M. sexta* that are optimal for growth.

**Figure 4:**
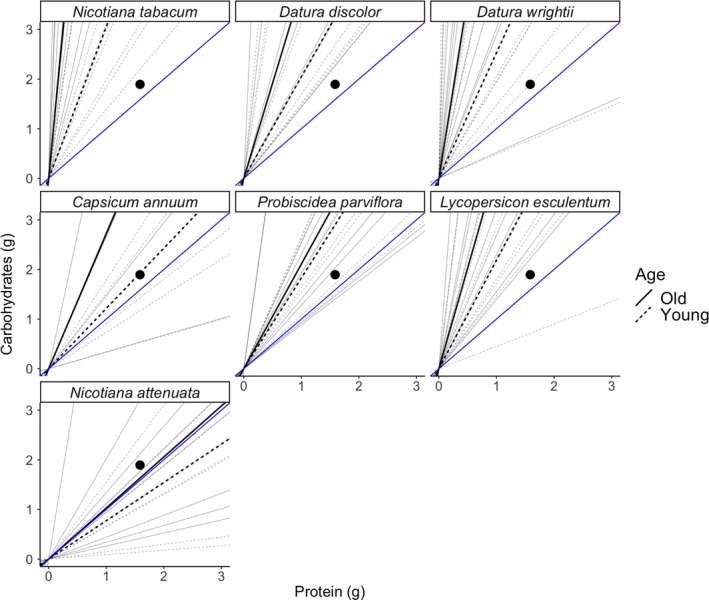
Nutrient rails of seven different host plants of *Manduca sexta*. Each faceted panel represents the nutritional space of a different host plant. The slopes of Blue lines represent the 1:1 ratio of protein to carbohydrates. Solid lines represent nutritional rails for old leaves whereas dashed lines represent nutritional rails for young leaves. Gray lines are raw values whereas black lines represent the median value for each group. The black dot represents the average target intake (1.587 g of carbohydrates and 1.894 g of protein) of a *M. sexta* larvae over their development (as measured by previously—Wilson et al., 2019). Rails to the left of the 1:1 P:C rail indicate that the larvae are protein constrained on that host‐plant species and rails to the right of the 1:1 P:C rail are carbohydrate constrained

We measured p:c ratios of individual leaves on multiple individuals from a given species—each leaf can be visualized in nutrient space as a rail—a line with a slope that is the p:c ratio (Simpson & Raubenheimer, 1995). *M. sexta* larvae move among different leaves on a single plant throughout development (Casey, 1976; Potter, Bronstein, & Davidowitz, 2012) and have leaves of all ages to feed on. In Figure 4, we plot the nutrient rails of both young and old leaves for all species, which illustrates a few key points about the system. First, we show in previous work that *M. sexta* caterpillars from a long‐standing colony in our laboratory need p:c ratios of approximately 1:1 for optimal growth (Wilson et al., 2019). When combined with results from the current study in Figure 4, a few key points emerge. Importantly, almost all plant species we measured demonstrated p:c ratios that were less than optimal (low in protein). Some plant species (*N. attenuata* and *C. annuum*) provide diet sources that would allow larvae to reach target intakes without substantial compensatory feeding, but other common host plants (*N. tabacum* and *D. wrightii*) typically have leaves that are relatively low in soluble protein, meaning that larvae would have to eat more leaf material to reach target values (Figure 4). These patterns suggest a mechanism that explains high reported leaf damage in some species (e.g., *N. tabacum* and *D. wrightii*; Casey, 1976; Potter et al., 2012; Wilson & Woods, 2015): Larvae are forced to eat extra leaf material to acquire enough protein, even on younger leaves with higher protein content (Figure 4), though this pattern may be complicated by the inclusion of secondary metabolites. However, previous work has shown that *M. sexta* is relatively tolerant to defensive compounds produced by plants in Solanacae (Wilson, Tseng, Potter, Davidowitz, & Hildebrand, 2018; Wink & Theile, 2002). Second, there are substantial differences in the nutritional environments available to larvae developing on different host species (highlighting the potential impact of oviposition site selection), but also the range of nutrient space available to larvae moving between young and old leaves. Work in other plant species has demonstrated large shifts in nutrient composition over longer developmental times (Deans et al., 2016), and the impact of lag time between when we sampled leaves and when plants were first transplanted echoes this pattern (Tables 2 and 3). Combined, these results suggest that while there are marked differences among old and young leaves and different host plants, the nutritional landscape is complex for both female *M. sexta* moths deciding when and where to lay eggs (Potter et al., 2012) and for developing larvae trying to maximize growth and other components of fitness.

Though growth and body size are often good proxies for fitness (Arnold, 1983), other physiological attributes can have consequences for survival and reproduction. Previous work has shown a strong link between different aspects of the insect immune system and nutrition (Cotter et al., 2010; Wilson et al., 2019). Our recent work on *M. sexta* showed interacting effects of protein and carbohydrates in experimental diets on both growth and five measures of immune function related to defense against parasitoids (encapsulation, phenoloxidase activity, prophenoloxidase activity, hemolymph protein, and hemocyte density) (Wilson et al., 2019). Though the interaction of carbohydrates and protein was often complex, diets with reductions in protein were generally detrimental to both growth and immune function. This suggests that there is a spectrum of host plant quality, which could result in differing growth rates, a range of body sizes, and variation in immune function in a population of *M. sexta* that uses multiple host plants. Interestingly, *M. sexta* raised on both *N. tabacum* and *Probiscidea lousianica* (a closely related species to the *P. parviflora* used in this study) had smaller final sizes, longer development times, and reduced fecundity when raised on *P. lousianica* (Diamond, Hawkins, Frederik Nijhout, & Kingsolver, 2010). These results are contrary to the expectations from our data (Figure 4), where *P. parviflora* appears to be a higher quality host plant than *N. tabacum* (closer to a 1:1 p:c ratio). This apparent contradiction draws attention to the complex nature of these interactions and highlights the idea that nutrition interacts with other plant traits (such as secondary metabolites) to affect herbivore fitness (Chen, 2008). Though *P. parviflora* may be nutritionally superior to *N. tabacum*, it also has physical defenses (sticky trichomes) that make it difficult to process, particularly for young larvae (Mira & Bernays, 2002), in addition to a suite of defensive secondary compounds that could affect *M. sexta* performance, immune function, and fitness. Ultimately, a given host plant is a combination of components including macro‐ and micro‐nutrients and secondary metabolites, so while there are clear differences among host plants in two axes (soluble protein and digestible carbohydrates), other axes that were not measured in this study could have significant impacts on herbivore performance and fitness.

Large‐scale spatial and temporal variation in host plant quality and *M. sexta* adaptation may also drive patterns of host use dynamics. For example, different genetic lines of *M. sexta* varied in their performance on *N. tabacum* and *P. louisianica*, with wild lines being better adapted for high performance on the novel (and presumably lower quality) *P. louisianica* (Diamond et al., 2010). It is still unclear whether *M. sexta* is locally adapted to different host species across its wide range, and though *M. sexta* is an incredibly well‐studied species (e.g., Kanost, Jiang, & Yu, 2004), surprisingly little is known about its population structure and connectivity. Spatial and temporal variation in host plant quality is also likely important in structuring interactions between *M. sexta* and its hosts. For example, localized variation in microclimate or soil chemistry can impact plant quality, both in terms of nutrient composition (Mankin & Fynn, 1996) and secondary metabolites (Koricheva, Larsson, Haukioja, Keinänen, & Keinanen, 1998; Wilson, Woods, & Kessler, 2018). Similar temporal variation (on a variety of scales) has been demonstrated across a number of species (Deans et al., 2016; Laitinen, Julkunen‐Tiitto, Rousi, Heinonen, & Tahvanainen, 2005; Liu et al., 1998; Meyer & Montgomery, 1987; Wells & Metz, 1963). We controlled microclimate and soil chemistry in this study (as plants were grown in a controlled greenhouse under common‐garden conditions), but some genetic variation is expected given that we collected seeds of wild species (*D. wrightii*,* D. discolor*, and *P. parviflora*) from populations across southeastern Arizona. Our results indicate that there are consistent differences among species in nutritional content, in spite of substantial variation among individuals within a given species.

In conclusion, we show that host plants used by a specialist insect herbivore show substantial variation in two of the main components of nutrition relevant to insect herbivores: soluble protein and digestible carbohydrates. Though we did not measure growth performance and immune function here, our previous work provides strong evidence linking variation in nutritional space to measures of performance. Ultimately, the differences in plant nutrition we describe here translate into vastly different nutritional landscapes afforded to insects consuming and developing on these plants, which may have far‐reaching effects on the evolution and ecology of insect behavioral strategies and plant defense. Furthermore, we show that across all the species we examined, younger leaf tissue typically has a higher percentage of soluble protein than older leaf tissue and that the combination of tissue ages on a given plant can provide a way for herbivores to mediate their diet on a single plant. Finally, though one species in particular (*N. attenuata*) had a nutrient composition that is of higher quality compared with other species (a more optimal p:c), it also has lower total nutrient content compared with other species, meaning that *M. sexta* larvae (and other insect herbivores) would need to eat substantially more leaf material to reach intake targets. This study is one of only a few that has used the geometric framework to examine the nutrient space of plants from an insect‐centric nutritional perspective (soluble protein and digestible carbohydrates), and the first to our knowledge to describe the nutrient space of a suite of host plants used by a single insect herbivore. By better understanding the physiologically relevant nutritional landscape provided to insect herbivores by host plants, we gain insight into the ecological and evolutionary dynamics that shape the interactions between these two key groups in terrestrial ecology.

## CONFLICT OF INTEREST

None declared.

## AUTHOR CONTRIBUTIONS

JKW and GD conceived experiments and the project. JKW, LR, and JD carried out experiments and analyses. JKW led the writing of the manuscript, with support from LR, JD, and GD. All authors contributed critically to the drafts and gave final approval for publication.

## Data Availability

All data and code for analyses (R scripts) are archived and available on Zenodo (https://doi.org/10.5281/zenodo.3420883).
